# Posterior Reversible Encephalopathy Syndrome Presenting as Stroke Mimic

**DOI:** 10.5811/cpcem.2017.1.30607

**Published:** 2017-05-09

**Authors:** Daniel Frick, Martin Huecker, Hugh Shoff

**Affiliations:** University of Louisville School of Medicine, Department of Emergency Medicine, Louisville, Kentucky

## Abstract

We present the case of a 33-year-old male with end stage renal disease presenting to the emergency department (ED) with headache, dizziness, and unilateral weakness. Initial concern was for ischemic or hemorrhagic stroke. Magnetic resonance imaging confirmed posterior reversible encephalopathy syndrome (PRES). The patient was treated appropriately and made a full neurologic recovery. PRES is an under-recognized diagnosis in the ED. As a stroke mimic, PRES can lead the clinician on an incorrect diagnostic pathway with potential for iatrogenic harm.

## INTRODUCTION

Posterior reversible encephalopathy syndrome (PRES) is a neurologic condition characterized by localized vasogenic edema primarily affecting the occipital and parietal lobes.^1^ While PRES is not yet completely understood, it is typically seen in the setting of common clinical scenarios: hypertension (HTN), cytotoxic medications, eclampsia, autoimmune, and systemic conditions.^2^ The most commonly presenting symptoms of this disease are headaches, seizures, altered mental status, and visual changes or loss. Encephalopathy can range from mild confusion to a severe decrease in level of consciousness.^3^ While the exact incidence of PRES is not known, reported cases exist in patients from 4–90 years old with predominance in female patients, with a mean age of 45 years.^4^,^5^ Due to its rarity and variable presentation, PRES is difficult to diagnose without radiographic imaging. A noncontrast computerized tomography (CT) of the head is of little benefit and ultimately magnetic resonance imaging (MRI) of the brain is necessary to demonstrate vasogenic edema and other pathognomonic findings.

PRES is often overlooked due to clinical features shared with many common conditions including ischemic or hemorrhagic stroke, migraine, seizure, hypertensive urgency, and reversible cerebral vasoconstriction syndrome. Such a misdiagnosis can result in inappropriate use of thrombolytic therapy or other more invasive procedures, ultimately delaying appropriate treatment. Therefore, in a potential stroke patient with vision changes or altered mental status, PRES must be considered.

## CASE REPORT

A 33-year-old male with uncontrolled HTN and end stage renal disease (ESRD) presented to the emergency department (ED) just prior to scheduled hemodialysis due to severe HTN. Initial blood pressure was 248/165 mmHg and the patient complained of a worsening headache and throbbing pain behind his eyes. He admitted to missing his last session of dialysis and his laboratory values were remarkable for a blood urea nitrogen (BUN) of 53mg/dL, creatinine (Cr) 4.67mg/dL, and serum bicarbonate (CO2) 21 mEq/L. Remaining serum electrolytes and hepatic function panel were normal. Symptoms were attributed to volume overload due to missed dialysis, and treatment with furosemide 40mg intravenous (IV) was initiated. Repeat blood pressure was 197/134 mmHg, and the patient was transported to the dialysis unit.

Upon completion of dialysis he remained hypertensive and immediately returned to the ED with severe headache, dizziness, and unilateral weakness. His blood pressure was 200/135 mmHg, heart rate 93 beats per minute, respiratory rate 22 respirations per minute, temperature 98.9°F. Physical examination was significant for pupils equal and reactive to light bilaterally with extra ocular movements intact, cranial nerves II–XII intact, [3/5] strength in the right upper extremity, [5/5] strength in left upper extremity, [2/5] strength in the right lower extremity, [5/5] strength in left lower extremity. Laboratory values showed the following abnormalities: BUN 39mg/dL, Cr 3.67 mg/dL, and brain natriuretic peptide 42500 pg/mL; serum electrolytes, hepatic function and troponin were normal.

Initial noncontrast head CT showed hypodense areas in the right lentiform nucleus and the medial bilateral occipital lobes concerning for acute to subacute infarct. Potential hypodense areas in the cerebellar hemispheres concerning for acute to subacute infarct could not be distinguished from artifact from adjacent bony structures. The patient’s unilateral right-sided weakness and CT findings brought stroke to the top of the differential diagnosis and the stroke team was consulted. The patient’s blood pressure was initially treated with one sublingual nitroglycerin prior to IV access, followed by 10mg intravenous labetalol. One hour after the patient’s arrival his blood pressure was 136/90 mmHg. The patient complained of continued headache and progressively worsening bilateral visual field deficits involving both inferior quadrants of each eye as well as bilateral loss of visual acuity. His blood pressure remained labile and he was subsequently placed on a nicardipine drip at 2.5mg/hr for more precise blood pressure titration.

Following blood pressure stabilization, an MRI brain without contrast (our institution’s stroke protocol) was obtained. MRI showed extensive T2 fluid-attenuated inversion recovery (FLAIR) hyperintensities involving the occipital lobe gray matter and the subcortical white matter of the occipital, parietal, and frontal lobes. T2 FLAIR hyperintensities were also present in the deep gray and white matter, periventricular white matter, cerebellum, midbrain, and pons. A mixture of cytotoxic and vasogenic edema was seen as diffusion-weighted hyperintensities involving the left body of the corpus callosum and an area of deep white matter in the posterior right frontal lobes (Image).

CPC-EM Capsule

What do we already know about this clinical entity?

Posterior Reversible Encephalopathy (PRES) is vasogenic edema of the brain resulting in a spectrum of neurologic deficits ranging from headaches and vision loss to seizures and encephalopathy.

What makes this presentation of disease reportable?

PRES is often overlooked due to clinical features shared with common conditions. If promptly diagnosed and managed, PRES can result in favorable patient outcomes.

What is the major learning point?

Missed or delayed diagnosis of PRES may result in improper administration of thrombolytic therapy and iatrogenic adverse events.

How might this improve emergency medicine practice?

Maintaining a broad differential in patients with neurologic deficits will allow physicians to accurately diagnose and appropriately treat life-threatening conditions.

The patient was admitted to the stroke service and was eventually transitioned to oral anti-hypertensive medications. His vision began to improve 24 hours after admission and upon discharge eight days later he had markedly improved visual acuity: 20/20 in the right eye with a mild right inferior quadrantopia and 20/40 in the left eye. His hospital course was prolonged by dialysis requirements and transfer to the psychiatric floor due to suicidal ideation and depression stemming from his poor health and hospitalization.

## DISCUSSION

PRES was initially described in 1996 by Hinchey et al. as a clinical and radiologic diagnosis most often presenting with headaches (50%), vision changes (33%), seizures (60–75%), and less commonly focal neurological deficits (10–15%).^6^,^2^ Our patient presented with focal neurologic deficits involving the right upper extremity and right lower extremity as well as dizziness, headache, and vision changes. Our initial presumed diagnosis was stroke, and this appeared to be confirmed with the noncontrast head CT showing multiple hypodense areas. While PRES can be suspected based on clinical presentation, diagnosis is often confirmed radiographically. A noncontrast head CT rarely aids in the diagnosis; however, a CT perfusion (CTP) study may show increased cerebral blood volume, cerebral blood flow, and reduced time to peak blood flow.^9^ In addition, hyperperfusion indicating high metabolic demand on CTP imaging may indicate seizure as a possible cause of neurologic deficits versus an acute ischemic insult.^10^ Such CTP findings can facilitate rapid diagnosis of PRES and other stroke mimics, allowing for quicker initiation of appropriate therapy. PRES commonly manifests on MRI as vasogenic edema in the parieto-occipital regions of both cerebral hemispheres with involvement of the subcortical white matter and the cortex.

Given diverse presentation, PRES has three primary variations: a dominant parieto-occipital pattern, holohemispheric watershed pattern, and superior frontal sulcus pattern.^7^,^8^ The diffuse intracranial involvement as seen on MRI in our patient appears to be an unusual presentation for PRES.

The vasogenic brain edema is believed to be the result of increased local perfusion pressure resulting in the failure of the blood brain barrier to maintain the compartmentalization of intravascular fluid. Capillary endothelial integrity is compromised due to increases in blood pressure, various systemic disease states, or cytotoxic medications. Blood pressure increase detected by cerebral endothelial mechanoreceptors leads to cerebral arteriolar vasocontricton. The autoregulatory vasoconstriction system of the arterioles becomes overwhelmed and leads to an increase in cerebral blood flow.^11^,^2^ If the hyperperfusion overwhelms the autoregulatory response, break down of the blood brain barrier can occur.

The relatively poor sympathetic innervation of the posterior fossa of the brain makes this area particularly susceptible to hyperperfusion and vasogenic edema.^2^ Our patient presented with multiple episodes of high blood pressure prior to the onset of his symptoms. This appears to have allowed ample time for disruption of the autoregulation of the cerebral vasculature, breakdown of the blood brain barrier, and ultimately vasogenic edema. While HTN is thought to be a large contributor to the development of PRES, 20–40% of patients are normotensive.^12^

Other pathophysiologic explanations include an inflammatory response affecting vascular permeability directly through cytokine release. Cytokines activate endothelial cells to secrete vasoactive factors, leading to increased vascular permeability and interstitial brain edema. It is postulated that autoimmune disorders can lead to PRES through a similar process of upregulation of cytokines and poorly controlled inflammatory state. PRES also occurs in patients taking immunosuppressive or cytotoxic drugs for malignancy or organ transplantation. Such medications can result in PRES immediately or several months after initiation of the drug, even when drugs are at therapeutic levels. This is thought to occur through dysregulation of vasoactive substrates such as tumor necrosis factor alpha and vascular endothelial growth factor. Probably least understood is the correlation between renal failure and the development of PRES (up to 55% of patients). Our patient’s long-standing hypertension in the setting of ESRD predisposed him to the development of PRES.^2^

The treatment of PRES is challenging and multifaceted. Experts agree on the importance of blood pressure control, though no studies have been conducted to establish causation or to correlate blood pressure management with resolution of PRES.^2^ Blood pressure reduction of 25% within the first few hours is recommended, but caution must be taken as the pressure is often labile. If PRES is attributed to a specific medication, the medication should be discontinued immediately. The risk and benefits of restarting the medication should be considered following the resolution of PRES.

The prognosis of PRES is typically good, with some sources citing 75–90% of patients making a full recovery with a mean time to recovery of 2–8 days. The most severe forms of PRES can result in death with a mortality range of 3–6% in 1–3 months.^2^,^3^ Severe neurologic injury and death typically result from intracranial hemorrhage, posterior fossa edema with brainstem compression, or increased intracranial pressure resulting from diffuse cerebral edema.^13^,^14^ In 10–20% of patients, long-term neurologic sequelae such as seizures, hemiparesis, decreased visual acuity, and residual dizziness have been noted.^15^,^2^,^16^ PRES is recurrent in 5–10% of patients with uncontrolled HTN as compared to other causes of PRES.^17^

## CONCLUSION

Though uncommon, posterior reversible encephalopathy syndrome should be considered in patients with new onset visual loss, especially when accompanied by headaches and altered levels of consciousness. Due to significant overlap with other neurologic disorders, PRES must be considered early and pursued with advanced radiographic imaging. Though often reversible, PRES can result in permanent brain injury and death if not recognized early and treated appropriately.

## Figures and Tables

**Image:**
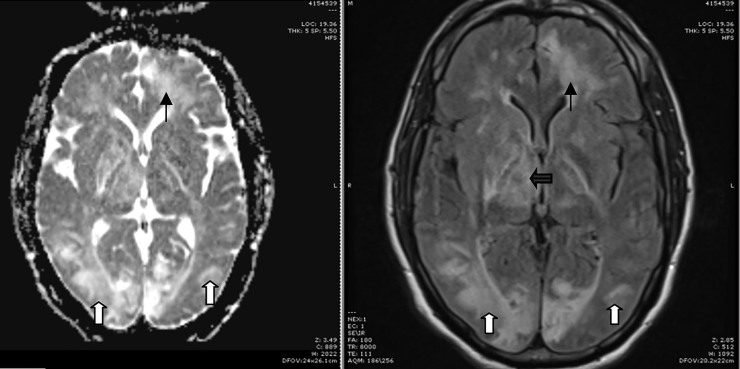
MRI Left: Diffusion imaging showing increased signal in the bilateral occipital lobes (white arrows) and left frontal lobe (black arrow). MRI Right: T2 FLAIR imaging showing correlating hyperintensities in bilateral occipital lobes (white arrows), left frontal lobe (black arrow) and right periventricular area (black bold arrow). *MRI*, magnetic resonance imaging; *FLAIR*, fluid attenuation inversion recovery
